# On the Endosmotic Action of Medicines

**Published:** 1852-02

**Authors:** 


*On the Endosmotic action of Medicines.*—Dr. Cogswell lately read a paper on this subject at the Medical Society of London. After some remarks on the construction of the apparatus—the properties of different membranes to be examined—the well known deductions of Poiseuille, in his Memoir in the "Comptes Rendus" of the French Academy of Sciences for 1844,—the author proceeded to mention his own observations. The endosmometer of Dutrochet consisted of a glass tube, with a somewhat bell-shaped moveable expansion called the reservoir, having a deep contraction round the middle for securing the membrane. The form of reservoir preferred by Dr. Cogswell was that of a bell-jar with a projecting rim round the larger orifice, the end of the tube and the inside of the reservoir being ground to fit one another. The reservoir had a capacity of eighteen drachms, and an internal diameter at the larger orifice of an eighth of an inch. The calibre of the tube was a fourteenth of an inch. To support the reservoir the tube was passed through a cork adapted to a hole in a leaden plate, which rested on the edge of the outer vessel. This was a glass cylinder, of such dimensions, that, on receiving the reservoir, a quantity of fluid, equal to the contents of the latter, would rise to the neck, leaving sufficient below the membrane. On consideration, the author had been led to adopt, for closing the reservoir, the cæcum of the sheep, as sold in a prepared state by the French, finding, in comparison with other membranes, that it produced the most marked results. The experiments of Poiseuille were then examined in the order observed in his Memoir.

*Action of Purgatives.*—Seidlitz water contained in the reservoir, being opposed to serum, ascended in the tube. Albumen was found in the reservoir, and sulphate of magnesia in the serum. Now, Seidlitz water causes an unusual quantity of albumen to appear in the alvine discharges, and of sulphate of magnesia in the urine. Hence the inference is, that this class of purgatives possesses the property of determining a flow of serum towards the bowels. The author remarked, that it might reasonably be questioned whether serum was a fair representative of the living fluid in the bloodvessels, or its accumulation in the bowels the only physiological effect of the saline purgatives.

*Tolerance of Medicines.*—The author remarked, that endosmose was found by Poiseuille to stop at periods varying for different fluids. The outer fluid being then examined, presents a striated appearance from the incomplete diffusion of the foreign matter introduced in it. After shaking it, there is a renewed ascent of the column; and the same thing happens repeatedly. Poiseuille employed a solution of phosphate of soda and serum. The author repeated the experiment with a solution of the salt, of density 1060, and obtained similar alternations, except as regards the elevation following the second employment of the serum. He left it to be judged, whether the facts as stated would bear out the inference, that the tolerance of medicines arises simply from the circumstance, that "the membranes of the intestinal canal, after being long in contact with the same substance, become impregnated with it, and prevent it from entering so freely into the circulation."

*Influence of Opium.*—Opium and its salts check diarrhœa, and obviate the purgative tendency of other medicines. A solution of one part of nitre to eight of water was opposed by Poiseuille to serum, and produced an elevation in the tube for three quarters of an hour. While the endosmose was proceeding vigorously, the solution was withdrawn, and replaced by a similar one, containing muriate of morphia. After this the ascent continued, but with less intensity; it proceeded for an hour, ceased an hour, and then the column began to descend. Hence, it is said, the presence of the morphia diminished the endosmose, then put a stop to it, and ended by producing exosmose, such being precisely its effect in promoting extirpation of the bowels. The author, however, believed, that if the experiment had been continued without the morphia, the result would have been nearly the same, as he had found that nitric by itself has but a feeble form of endosmose. To ascertain further whether opium exerts a peculiar influence on membranes unfavorable to endosmose, he had repeatedly opposed an aqueous solution to water, and from it produced much greater effect than some of the inorganic salts. The serum of the sheep inclosed in a reservoir, and opposed to distilled water, containing a grain to the ounce of muriate of morphia, produced a vigorous endosmose for above twenty-four hours. Added to syrup in the same proportion, its effect was not appreciable. He was thence led to believe there was not sufficient ground for characterizing morphia as a substance the presence of which puts a stop to endosmose, and renders the membrane impermeable to either fluid.

*Influence of Tobacco.*—The decoction of tobacco is stated, by M. Poiseuille, to penetrate the membrane, and render it unfit for endosmose. A decoction of four parts of tobacco-leaves to forty of distilled water, was opposed to serum. There was a descent of the column in the tube. However, the density of the two fluids was not stated. The author having made a similar decoction, found that after boiling above an hour, the density did not exceed 1023, when it was not likely to produce endosmose, with serum having a density of probably not less than 1026. But a decoction of this strength, being opposed to distilled water, produced an elevation lasting for several hours; and further, a decoction of density 1052 opposed to serum of density 1031, produced a well-marked elevation of the column, which was found not to have stopped in twenty-one hours. The author proceeded to state, that having observed a great variety in the endosmose afforded by different solutions of the same density, he tried the following experiment:—Four endosmometers, closed with the prepared cæcum, were filled respectively with solutions of sugar, sulphate of magnesia, common salt, and nitrate of potash, and placed in distilled water; in half an hour the first fluid ascended 1·9 inch, the second, one inch, the third, 2 inches, and the fourth, ·8 of an inch; other membranes afforded corresponding, though less marked results. Thus the common salt was the most energetic at first, and the nitre the least so. But again, the syrup and sulphate of magnesia continued to ascend for several hours, while the common salt stopped in four hours and the nitre in less than two. Syrup, though it has a remarkable power of endosmose, is not a purgative, which Poiseuille accounts for by its being decomposed by the gastric juice. The author then extended the examination to classes of substances. The results obtained were arranged in a tabular form and laid before the Society. It was remarkable, that the sulphates from which experience has selected the most generally useful purgatives, had invariably a strong and continued action, while the class to which nitre belonged was comparatively feeble. Chlorate of potash and the iodide and bromide of potassium were among the substances which had the lowest place in the table. Gum and liquors showed a moderate degree of energy, but it continued uninterruptedly for weeks. The author, after entering into some further details, said, he mentioned these as coincidences which might prove useful aids to investigation, but without any view to the formative construction of a theory. From what proceeded, he was lead to the following conclusions:

1. That the division of substances into those which are favorable to endosmose, and those which on the one hand retard and annihilate it, by their influence on the membrane, and on the other render the membrane permeable, or reduce it to the condition of a filter, requires confirmation.

2. That the power of endosmose of different solutions is not regulated entirely by their density, as already observed by Dutrochet.

3. That the purgative salts generally have an energetic form of endosmose, and that this is exerted with more steadiness and uniformity by those which medical experience has selected as most useful in ordinary circumstances.

4. That some of the other substances have marked peculiarities with regard to endosmose, which will probably assist towards explaining the mode of action on the system.

Dr. Lankester thought that the Society and the profession were indebted to Dr. Cogswell for his original and interesting paper. It might not be regarded as practical; but many of the theories discussed by the author had resulted in practical applications. Investigations which proved the erroneousness of old theories were as valuable as those which established new ones. The practical man always has a theory to explain his practice; however disagreeable it was to give up an old theory with its resulting practice, all progress in science and art demanded it. The investigation of the physical properties of matter had contributed greatly to our knowledge of the functions of life; and imperfect as was our knowledge of those properties of membranes called exosmose and endosmose, it had opened a field of useful inquiry. Dr. Cogswell's experiments showed at least that the theory which explained the action of saline purgatives by their increasing endosmose, was only partially true. They might, however, contribute to the explanation of the action of other medicines. The extraordinary endosmotic power of acetate of ammonia was worthy of remark; and whilst it showed that this power was not alone the cause of purgation, it might throw light on the action of that medicine. He was somewhat surprised at Dr. Cogswell's conclusions with regard to morphia, as the experiments of Poiseuille and Bachetti went to prove that it diminished and even reversed the endosmotic action of fluids in which it was contained. This explanation of its action in diarrhœa was thus rendered valueless. It should, however, be recollected, in reasoning from phenomena occurring out of the body to those taking place within it, how different were the conditions. In the human stomach and intestines we had a living surface covered with cells in a constant state of developement, and with mucus, which must necessarily modify any endosmotic action, even were that the most prominent function of these tissues. If Dr. Cogswell's paper only led to negative results, it would be valuable as indicating the necessity of caution on a subject on which there had been a good deal of positive speculation.

Dr. Handfield Jones said, that in the paper just read there were two points to which he wished to refer. First,—Certain membranes of a homogeneous character altered the nature of the fluids which passed through them. In illustration of this he instanced the Malpighian tufts of the kidney, and the membrane of the aqueous humor of the eye. This fact was interesting in respect to endosmotic action, which was known to be so greatly modified, in many instances, by the kind of membrane employed. Second,—He referred to the case of the secretion from the kidneys, in which the blood containing the elements of the secretion was on one side of the homogeneous basement membrane, and a layer of albuminous semi-solid matter in the form of epithelium on the other; and inquired whether the elimination of the secretion might not be regarded as an endosmotic action.

Dr. Snow said that, although the subject of endosmosis was a very important one, it did not go far to explain the action of medicines, even of the purgative class. It might occasionally assist in the action of some of the saline purgatives, as Epsom salts, but this medicine would purge when given in repeated small doses, and so diluted as to be much less dense than the serum of the blood. One important point, which it was requisite to bear in mind respecting endosmosis, had been stated by Dr. Golding Bird—viz., that acetate of potash and other salts, when intended to act as diuretics, must be sufficiently diluted to enable them to be imbibed, otherwise they would set up endosmosis into the interior of the alimentary canal, and act as cathartics. Opium probably prevented purgation by diminishing the peristaltic action of the intestines. The theory of its diminishing the permeability of animal membranes would not explain its power of checking diarrhœa, even if correct; for the absorption of fluids taken into the alimentary canal would be retarded, and this would have the opposite effect. In order to understand the action of medicines, a number of other laws must be taken into consideration besides those of endosmosis.

Mr. Chippendale commended the manner in which Dr. Cogswell had conducted his experiments, but thought he had not succeeded in showing that the action of inorganic salts, as purgatives, was the result of endosmosis. In the first place, the fluid found in alvine evacuations was not serum, and if it were a transudation of fluid, as the result of endosmotic action, we should naturally expect that it would take place chiefly through the coats of the stomach, and gradually become less along the course of the alimentary canal. Experience, however, had shown us that purgative salts act principally in the colon. Besides, if serum were really to pass through the coats of the alimentary canal by a process of endosmosis, it would be constantly going on, for the mucus which lubricated the internal surface of the tube was denser than serum. As a striking instance against the view that purgative action was one of mere endosmosis of serum, he illustrated what took place when a dose of castor-oil was taken. He considered, in conclusion, that purgative salts did not act by causing an endosmosis of serum, but that probably the epithelial cells had much to do with the matter.—*London Lancet.*

